# Recreation in Relation to the Health of the People

**Published:** 1891-08-15

**Authors:** 


### Recreation in Relation to the Health of the People.

### VII.—AMONG THE MINING POPULATION.

It was urged in our last article that those who devote themselves to the task of trying to make the lives of the people brighter and better, gave their attention too exclusively to London; and the corollary which followed upon that proposition was this: that more attention should be paid to country people in the matter of recreation. There might well have been another corollary, however—namely, that our mining population also needed consideration; indeed, we do not know but what this wants stating with even more emphasis than the other. True, there is no question here, as there is in our work in the country, of making the life with which we are concerned sufficiently attractive to compete with London life. So far as we know, there is no tendency among miners to "chuck" their work and migrate to London; their present life is not without excitement, and gives them plenty of fellowship, and, therefore, is not felt, like the country labourer's life, to be slow and dull. But for some reasons it is more important even to provide recreation for them than for him. Granted that the labourer has a quiet and often monotonous life, still he works all day in the light of heaven; the pure, fresh breezes play around him; and a thousand and one little interests spring up about him, apart from or alongside of his daily work. A lark's nest lies *perdu* in the grass he is about to cut; a flight of wild ducks speckles the blue above him; a flock of sheep—not from that neighbourhood, he knows—comes trooping down the dusty road; Reynard the fox scurries by, and gleams of white and scarlet in the distance tell him that, almost any minute, a whirlwind of hounds, horses, and men may follow, with yelp and holloa. An intelligent labourer is full of general knowledge of a limited kind (if we may be pardoned the bull); and although, alas! he is often *not* intelligent, he always has the materials upon which his intelligence, if once set going, may work.

But to come back to the miner. How does his life compare with that of the countryman? Like a mole, he burrows beneath the earth, instead of working under the open sky; for sunshine, he has the light of his lantern, for the winds of heaven, a heavy atmosphere which oppresses and sometimes well-nigh chokes him. As for interests springing out of his work, yet apart from it, such as the country labourer has, or may have, in plenty—well, we do not see what study the miner has facilities for taking up, unless, indeed, it be geology. Somewhere in the bowels of the earth there may be a miner who, while "digging dusky diamonds," dreams of the primeval forests which once reared tall heads into the warm steaming atmosphere of which his geology books tell him, but if such a man there be, we, for our parts, have yet to find him. No wonder—to come back from wild fancies to sober facts—no wonder that the miner, when once he emerges from his subterranean workshop, seeks recreation before turning into his home—often only a few degrees less gloomy and grimy than the mine.

But does the miner, then, seek this recreation in vain? By no means. There is always the drink to fly to. When money is plentiful, he often indulges himself in most expensive liquors, and when it is scarce he can still turn to his favourite "clink," which is as dangerous as it is cheap. We believe it to be beer worked up again with sugar; but let not over-much confidence be placed in this statement, for we confess that here we copy the action of the immortal "Brick Lane Branch" Committee with relation to "dog's nose," and state this thing upon hearsay, not upon personal knowledge.

The miner has his drink then—who but he? Moreover, he has his betting; and a very engrossing form of recreation this appears to be. He flies pigeons, and bets upon the result of the race. He keeps dogs, training them to race at the wave of a handkerchief—"sprinting," we believe it is called; and here again the betting is the most important part of the proceedings, the miner taking so keen an interest in the race that, when food is short, he will often stint his wife and children, rather than his dogs. Bull-baiting was formerly another of the miner's recreations, dogs being kept and trained for this purpose also; and until quite lately, stand-up fights—not from bad blood, but just for the love of the thing—were very frequent, and attracted large and admiring crowds of spectators.

Football? Yes, this is a form of recreation greatly in favour among the miners; but unfortunately it is valued, not for its intrinsic worth, but for the opportunities which it affords of betting. This excellent game, instead of being, as it ought to be, an outlet for the energies and spirits of the young miners as a body, becomes, in fact, a profession, such young fellows as show a special aptitude for it being encouraged to take to it for a livelihood, and supported by the betting clubs which those who were once his fellows are eager to join. It is, indeed, lamentable to see the straits into which, in our Midland districts, this noble game has fallen. What can be done to rescue it, we do not see, any more than we can see how horse-racing is to be divorced from its now universal concomitant—betting; but if anybody can set his wits to work, and devise plans for accomplishing this purpose, he will be doing good service to the mining community.

We have now arrived at the sum total of the miner's recreations, unless, indeed, we include wife-beating, in which certain miners indulge, and which may perhaps be regarded by them in the light of a pastime, akin to the coster's "jumping on his mother." It is obvious that there is a wide field for work, and that the work urgently needs doing; but when we come to the question, What is done in the way of providing wholesome recreation for our miners?—echo answers What? The clergy in our large mining towns are fairly swamped, as we may say, by the streams of puddlers, shinglers, miners, and pit-girls, which pour forth from the iron furnaces, the pit and the pit-mouth, crying out, though they know it not, for help—for wholesome food for the mind and for the soul. For the latter the clergyman strives, yet strives in vain, to provide sufficiently; for the former he can do practically nothing. Sometimes, indeed, he does make an effort, and a successful effort, to provide for those who are drawn by his teaching, and have a special claim upon him for help; but this is as a mere drop in the ocean. As one energetic and earnest clergyman pathetically remarks: "What we want to do is to get hold of these fellows in the mass, not of merely a few well-meaning ones, who might probably keep fairly straight without us. But how to do it, with no time, no money, no help—there's the rub." Ay, there's the rub. Will no one come forward to try what bright and attractive coffee-palaces, which should provide not only coffee and newspapers, but amusements, would do for our miners? Such palaces answer well in other towns—in Leicester, for instance—why should they not answer here?

Again, miners are musical; let us put that fact in our pipe, and see whether the suggestions which issue therefrom do not end in something better than smoke. Excellent ears and excellent voices are to be found in plenty among the mining population; and if a *Messe Solennelle* in church can attract, as it does attract, a large mining congregation, it may reasonably be expected that concerts and choral classes may also meet with success.

Here is the Need; where is the Man?

